# Supplementary figures and images for: Ozone ultrafine bubble water sterilizes Porphyromonas gingivalis and neutralizes its virulence factors
Source: PLoS One. 2025 Oct 14;20(10):e0334259. doi: 10.1371/journal.pone.0334259 (PMC12520332; doi:10.1371/journal.pone.0334259)

A

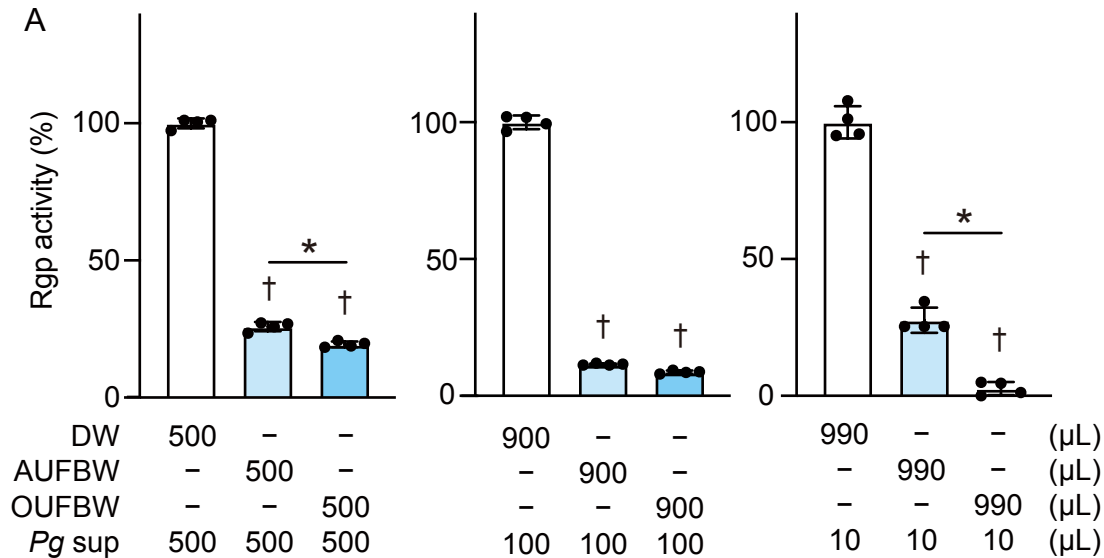

B

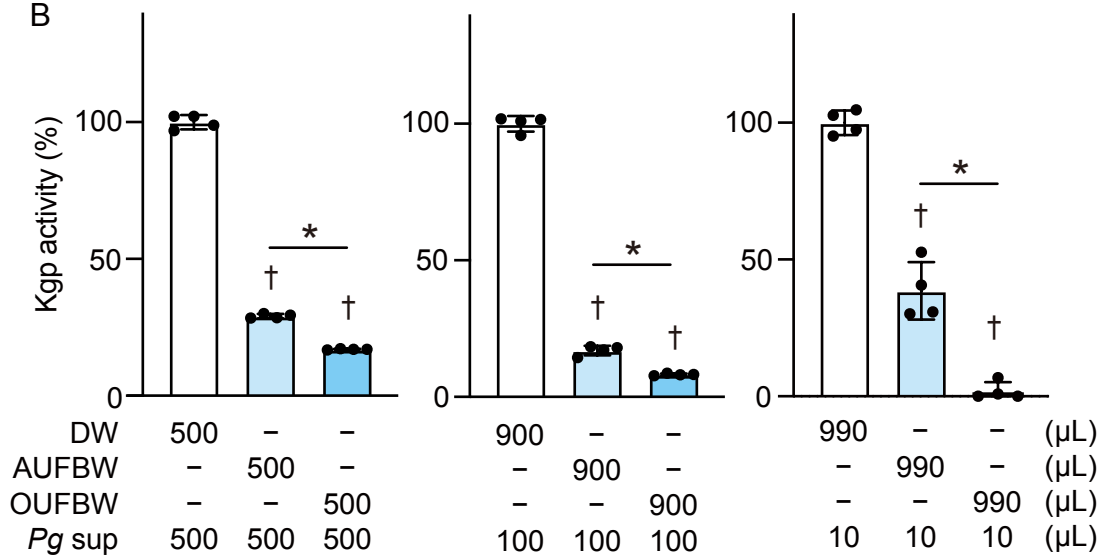

Supplement: S1 Fig — P. gingivalis culture supernatant (Pg sup) was exposed to distilled water (DW), air ultrafine bubble water (AUFBW), or 4.14 ppm ozone ultrafine bubble water (OUFBW) for 30 s. (A) Rgp and (B) Kgp activities were determined using Rgp- and Kgp-specific substrates, respectively. The values of the OUFBW and AUFBW groups were normalized against that of the DW group. The data represented the means ± SD of quintuplicate experiments and were evaluated using one-way analysis of variance with Tukey’s multiple comparisons tests. †, significant difference compared with the DW group at P < 0.05. *, significant difference between the indicated groups at P < 0.05. (PDF) [file pone.0334259.s001.pdf]

Fig 4A

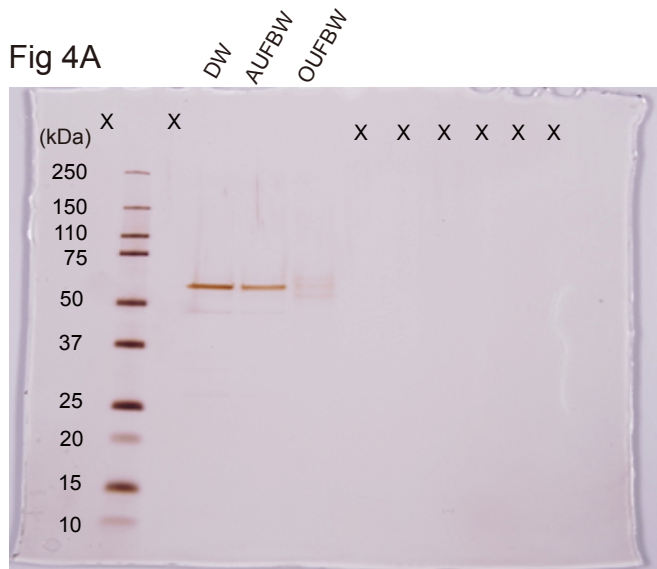

Fig 4B

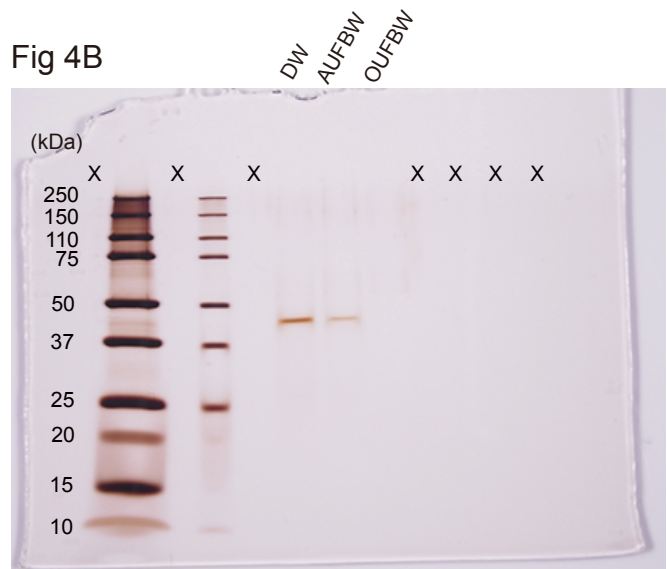

Fig 4C

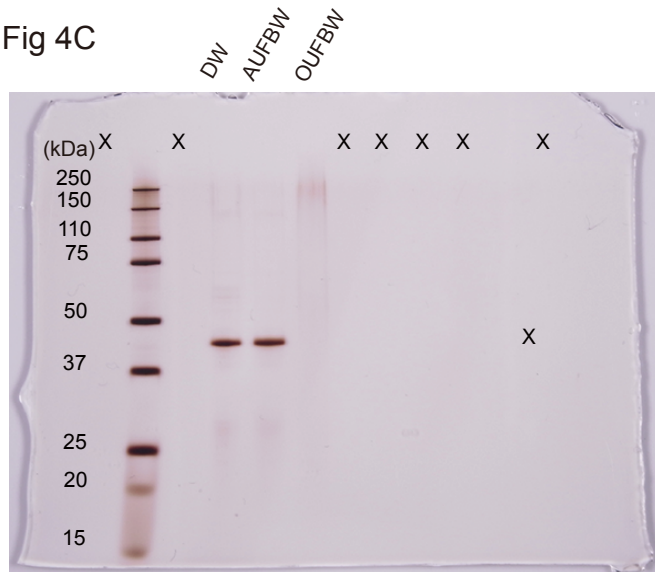

Supplement: S2 Fig — Untreated silver-stained images of Figure. 4A–C are shown. Images were obtained by scanning the gel using an image scanner. (PDF) [file pone.0334259.s002.pdf]

Fig 4D

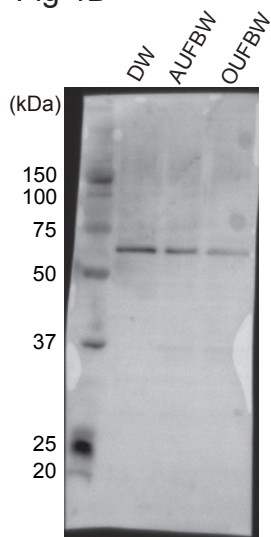

Fig 4E

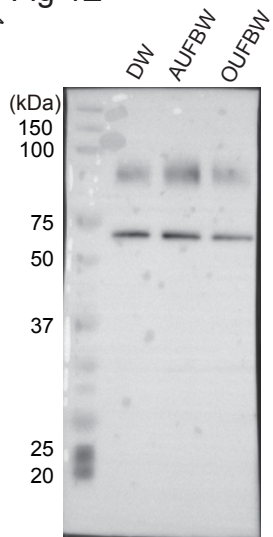

Fig 4F

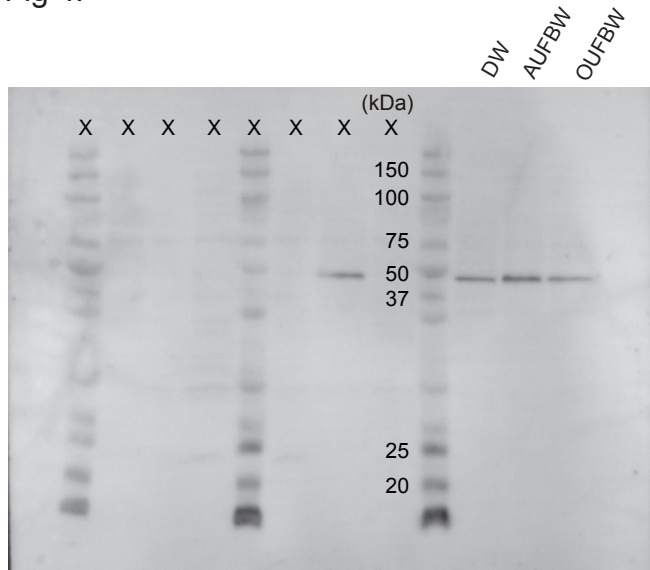

Fig 5A

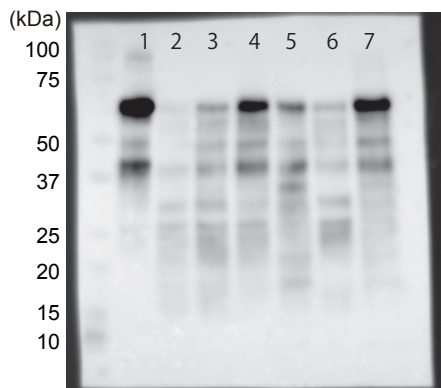

Fig 5B

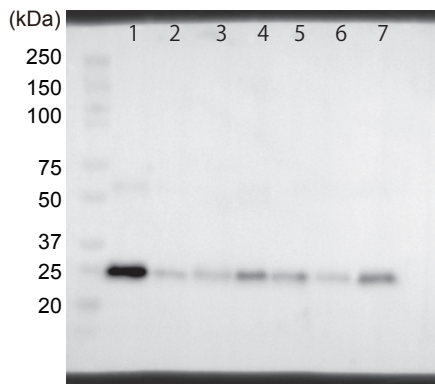

Supplement: S3 Fig — Untreated western blotting membranes are shown. The area framed in red are shown in each figure. (PDF) [file pone.0334259.s003.pdf]
